# Mpox Illness Narratives: Stigmatising Care and Recovery During and After an Emergency Outbreak

**DOI:** 10.1177/10497323241234482

**Published:** 2024-03-10

**Authors:** Anthony K. J. Smith, Daniel Storer, Kari Lancaster, Bridget Haire, Christy E. Newman, Sara Paparini, James MacGibbon, Vincent J. Cornelisse, Timothy R. Broady, Timmy Lockwood, Anna McNulty, Valerie Delpech, Martin Holt

**Affiliations:** 1 Centre for Social Research in Health, 7800 UNSW Sydney, Sydney, NSW, Australia; 2 Australian Human Rights Institute, 7800 UNSW Sydney, Sydney, NSW, Australia; 3 School of Population Health, 7800 UNSW Sydney, Sydney, NSW, Australia; 4 Kirby Institute, 7800 UNSW Sydney, Sydney, NSW, Australia; 5 Goldsmiths University of London, London, UK; 6 SHARE Collaborative, Wolfson Institute of Population Health, 4617 Queen Mary University of London, London, UK; 7 6079 NSW Health, Sydney, NSW, Australia; 8 379373 Sydney Sexual Health Centre, Sydney, NSW, Australia; 9 379373 North Coast Population and Public Health Directorate, Port Macquarie, NSW, Australia

**Keywords:** mpox, biographical disruption, gay and bisexual men, public health, sociology, stigma

## Abstract

In May 2022, a global outbreak of mpox (formerly monkeypox virus) affected thousands of mainly gay and bisexual men. Mpox is usually a time-limited illness that can involve fever, pain, and skin lesions, but may require hospitalisation. There is scant research into the firsthand experiences of people affected by mpox, including experiences of symptoms, healthcare, and recovery. This study considers the different illness narratives of people who experienced mpox in Australia in 2022. In-depth interviews and 6-month follow-up interviews were conducted with 16 people, including 13 people diagnosed with mpox and three close contacts. All participants were cisgender gay or bisexual men living in Australia. Participants’ accounts described minor to severe periods of sickness, negative and stigmatising experiences engaging with healthcare, and some participants experienced long-term effects on their sexual well-being and complications from mpox. The emergency outbreak context meant that mpox was highly distressing, making it difficult to manage and producing varying forms of disruption to everyday life. Mpox was narrated as disruptive in different ways: as a minor interruption to holiday plans, a prolonged period of poor health, or a biographically disruptive event prompting a re-evaluation of sexual values and health. This analysis demonstrates that an unfamiliar emergent disease outbreak related to sexual practices and sociality can reconfigure personal life and sexual well-being, suggesting a need to focus on providing quality patient care in outbreaks of mpox and other infectious diseases.

## Introduction

In 2022, a global outbreak of mpox (formerly known as monkeypox virus) was declared a Public Health Emergency of International Concern (World Health Organization, 2022). Mpox is a zoonotic disease related to smallpox, first detected in humans in 1970 in the Democratic Republic of the Congo (Marennikova et al., 1972). Since then, human-to-human transmission of mpox has primarily affected people in West and Central Africa (Adetifa et al., 2023). Mpox is characterised as an acute and self-limiting disease with a varying fatality rate (less than 0.1% in 2022 and between 1% and 12% in past outbreaks; Mitjà et al., 2023), and it has the potential for serious complications and hospitalisation. Common symptoms include fever, pain, and skin lesions, as well as other complications such as secondary bacterial infections, severe scarring, bronchopneumonia, and ocular issues (Mitjà et al., 2023).

The 2022 global outbreak resulted in over 88,000 cases and 152 deaths, primarily in Europe and North America, and the first time sustained transmission has been observed outside of Africa (Centers for Disease Control and Prevention, 2023). In Australia, 147 mpox notifications were recorded in 2022, with no fatalities, and the majority of cases were acquired by gay, bisexual, and other men who have sex with men travelling overseas, with limited local transmission (Cornelisse et al., 2023). The status of mpox as a public health emergency was rescinded by the World Health Organization in May 2023 after case numbers fell substantially (World Health Organization, 2023c). Transmission in the 2022 outbreak was reported to be primarily associated with sexual activity between men, in contrast to past outbreaks in which household transmission was most common, including through respiratory and fomite transmission (Mitjà et al., 2023).

The rapidity of the 2022 outbreak, its previous name (monkeypox) and endemicity in African countries, its apparent links to sexual transmission, and the recency of COVID-19 resulted in heightened public interest and media coverage of mpox. This (re)produced discriminatory tropes about gay and bisexual men and racial minorities (Damaso, 2023; Garcia-Iglesias et al., 2023; Gonsalves et al., 2022; Owens & Hubach, 2023; Paparini et al., 2023). Media stories included reports of patients experiencing stigma and discrimination in healthcare, difficulties with self-isolation and accessing treatment, inadequate pain management (Ryan, 2022a), and long-term effects of mpox, including scarring from lesions (Ryan, 2022b). A large multi-country survey of gay, bisexual, and other men who have sex with men identified a similar range of experiences (World Health Organization, 2023b). However, there appear to be no published firsthand qualitative accounts of illness and care related to mpox from the 2022 outbreak.

This study investigated the experiences of people closely affected by mpox in 2022, including their experiences with healthcare and how mpox disrupted life and a sense of future. Two key research questions guided the study: ‘What are the social and health experiences of people affected by mpox?’ and ‘How can people with mpox be better supported in healthcare?’

### Illness Narratives

Illnesses can cause a spectrum of life disruptions, from suspensions of ordinary routines and activities to major shifts in the experience of the self and expectations of the future (Hydén, 1997). Attending to people’s firsthand accounts of illness is valuable for generating insight into how particular illnesses are experienced, how priorities may or may not align with those of healthcare institutions, and revealing unexpected and unanticipated dimensions of illness that are not captured in clinical settings or surveillance systems (Timmermans, 2013).

In studying illness narratives, sociologists have long found value in applying the concept of ‘biographical disruption’ to understand how chronic illness and disability can dramatically reconfigure the way life is expected to be lived and the quality of social relations with others (Bury, 1982; 2001; Wells et al., 2023). In this conceptual model, chronic illness can threaten the existing, normative “relations between body, mind, and everyday life,” thereby producing an altered lifeworld (Bury, 2001, p. 264). The experience of illness and suffering is fundamentally relational, with illnesses having multiple potential effects on individuals, personal networks, and broader communities (Persson et al., 2019).

While the extent to which illness may transform life is well understood in the study of chronic illness, less attention has been paid to biographical disruption and acute illnesses. Defined by their time-limited (and self-limiting) potential, an acute illness nonetheless constitutes a temporal rupture in lifeworlds (Rosenfeld, 2006). For Rosenfeld, the distinction between ‘chronic’ and ‘acute’ illnesses is problematic, as it reifies a binary distinction between ‘ill versus healthy bodies’ and neglects the potential of illnesses with a short duration to generate lasting biographical disruptions. Rosenfeld (2006) argued that chronic and acute illnesses potentially share many similarities: disruption (of differing durations), distress (including uncertainty as a source of distress), and shifts in self and lifeworld that may persist despite the alleviation of symptoms and ill health.

In the case of acute infectious diseases, even if the period of symptomatic illness has passed, there may be enduring long-term effects of the disease, impacts on well-being, and social consequences such as stigma linked to the way the infection was acquired and fears around contagion. Several studies and commentaries have noted the role of stigma, blame, and shame (related to acquisition and the visibility of symptoms) as hindering responses to mpox and making it more difficult to engage in healthcare (Scheffer et al., 2023; Shrewsbury, 2022; World Health Organization, 2023b).

Many of the above issues observed with mpox are consistent with other illnesses associated with sex and desire, including HIV and sexually transmissible infections (Garcia Iglesias, 2022). Illnesses associated with sexual acquisition and other supposedly ‘deviant’ practices are often implicated by contested discourses about sexual morality and religious and cultural values (Sontag, 2002; Treichler, 1999). For example, the diagnosis of a sexually transmissible infection is often an occasion for a moral reinterpretation of self and can involve feeling ‘dirty’ and ‘guilty’ (Cook, 2014; Holt et al., 2010; Smith et al., 2022). The attention brought to genitalia and the rectum as a result of anogenital mpox symptoms potentially intensifies experiences of stigma (Ryan, 2022a). A lack of medical education regarding health issues affecting the penis and rectum, and understanding the rectum as a culturally conflicted site of pleasure, can exacerbate stigma when addressing colorectal health issues (Bersani, 1987; Halperin, 2009; Race, 2016; Vytniorgu et al., 2022).

Drawing on illness narratives and understandings of the dynamics of sexually transmissible infections from repeat interviews with people affected by mpox in Australia, this study sought to attend to both the ‘acute’ stage of mpox and its long-term effects and consequences.

## Methods

We conducted qualitative in-depth interviews with people diagnosed with mpox and close contacts in Australia (Smith, Storer et al., 2023). An initial sample was recruited from a national survey about mpox conducted in August–September 2022 (MacGibbon et al., 2023). Survey respondents who reported a diagnosis of mpox were invited to be interviewed if they had consented to be contacted. Snowball sampling from this initial sample (i.e. asking interviewees to share the study with contacts they knew had been affected by mpox) recruited additional participants diagnosed with mpox, along with some close contacts. Eligible participants lived in Australia, were aged 18 years or older, had the capacity to participate in an interview in English, and either had mpox in 2022 or were a close contact. Participants were offered compensation of 50 AUD for their time. Our study was approved by the UNSW Human Research Ethics Committee (HC220484) and the ACON Research Ethics Review Committee (202214). All participants provided written or verbal consent prior to interviews.

Initial interviews were conducted by A.S. between October and December 2022, at a time when the number of mpox cases had reduced significantly and public concern had abated, but experiences with infection were still recent. We also conducted 6-month follow-up interviews with those who consented to a second interview between April and May 2023. The purpose of follow-up interviews was to document any long-term effects or consequences of mpox, to allow further reflection on the illness event, and check interpretations of issues raised in the first set of interviews. Interviews were conducted by videoconferencing, by telephone, or in person, based on participants’ preference and location. Participants were asked about their experiences of mpox illness and diagnosis or being a close contact, diagnosis, isolation, care, recovery, disclosure, support networks, long-term effects of mpox, and reflections on the illness event over time. Participants were asked demographic questions at the end of the first interview, including their gender (current and sex reported at birth), sexuality, age, state of residence, country of birth, and ethnicity. Participants chose or asked the interviewer to nominate a pseudonym. On average, initial interviews lasted 70 min (range 46–120) and follow-up interviews lasted 40 min (range 18–58). Interviews were audio-recorded and professionally transcribed, and transcripts were checked for accuracy, de-identified, and organised for analysis with the assistance of NVivo software. Given the small number of mpox notifications in Australia, the author team have taken extra care to anonymise participant details.

We interviewed 16 participants in total. This included 13 people diagnosed with mpox (11 of whom participated in 6-month follow-up interviews) and 3 close contacts. This sample includes approximately 9% of all notifications in Australia in 2022 (*N* = 147). Diagnosed participants were primarily cisgender men and gay (with two bisexual or pansexual men) and were aged 25–56 years old (mean 43 years). The majority of participants were born in Australia (*n* = 5), followed by Aotearoa (New Zealand) (*n* = 2), the United Kingdom (*n* = 2), and one each from Western Europe, Canada, the United States, and South Africa. All participants reported Anglo or European ethnicities. Most participants lived in the Australian states of New South Wales (*n* = 7) and Victoria (*n* = 4), with one each from Queensland and South Australia. One participant diagnosed with mpox was living with HIV (on treatment and with an undetectable viral load), and all other participants reported a HIV-negative status and used HIV pre-exposure prophylaxis (PrEP). Participants received their mpox diagnosis during July (*n* = 8) and August (*n* = 5) 2022, and the majority reported acquiring mpox overseas (*n* = 11), with two acquiring mpox in Australia. Most participants (*n* = 8) underwent isolation or received care in Australia, while five did this overseas. Two close contacts were partners, and one was a housemate. All three close contacts were cisgender gay men.

Our approach to analysis was underpinned by principles of reflexive thematic analysis (Braun & Clarke, 2019). Following each interview, A.S. wrote fieldnotes, summarising key topics discussed in the interview and emerging analytical ideas. Fieldnotes informed updates to the study team and guided the conceptualisation of data. In between initial and follow-up interviews, A.S. and D.S. iteratively developed a coding framework based on research questions and reading interview transcripts, and D.S. refined this through the process of coding. D.S. wrote descriptive summaries of codes, and these informed the generation of initial themes. Themes were developed as shared patterns of meaning which aimed to convey common and distinctive experiences of illness and care, along with generating insight into the personal and social consequences of mpox diagnosis over time. Through these themes, this analysis tells a ‘story’ (Braun & Clarke, 2019) about mpox diagnosis and recovery, and the long-term impacts of mpox for some participants. Multiple workshops were also held with the broader author team to discuss interpretations. Follow-up interviews were conducted at this time and were an opportunity to learn more about participants’ experiences over time and hear how they re-narrated the initial illness event. In addition to noticing similarities between our data and journalists’ reporting (Ryan, 2022a, 2022b), the concepts of illness narratives and biographical disruption were useful in conceptualising participants’ accounts (Bury, 2001; Hydén, 1997; Rosenfeld, 2006; Wells et al., 2023). Our approach to analysis was both inductive and deductive; it was inductively developed through attending closely to participants’ experiential accounts, but also deductively informed by broader literature, concepts, and observations regarding mpox in other contexts. This analysis focused on providing insight into people’s firsthand experiences of mpox, how the events of illness and healthcare encounters were narrated, and the varieties of long-term effects generated by mpox illness.

## Findings

Participants described the period in which they were diagnosed as highly distressing. Mpox was an unfamiliar disease, and healthcare settings appeared to be underprepared to effectively test and diagnose it. Episodes of illness and required periods of self-isolation were described as difficult. The linking of mpox with sexual transmission and sex between men amplified the potential for stigma (Gonsalves et al., 2022). At the time participants were diagnosed with mpox, it had not yet been declared a Public Health Emergency of International Concern, but most participants were aware of mpox due to coverage on social media. Half the diagnosed participants were travelling overseas when symptoms appeared and when they were diagnosed, and mpox therefore disrupted travel plans and accommodation.

It was against this backdrop of ‘emergency’ and uncertainty that participants narrated stories of profound sickness and negative experiences of care. Beyond the early, acute stage of illness and care, some participants also described longer-term effects of mpox (including fatigue, anal complications, scarring, and poor mental health) and changes to values, relationships, and well-being. Our findings are presented in three themes. The first theme focuses on participants’ varying experiences of illness and long-term effects. The second explores two contrasting narratives about care. The third theme examines the biographically disruptive aspects of mpox, including how it produced changes to personal values and sexual well-being, and the degree to which mpox featured in participants’ lives over time. Taken together, these themes provide insight into a variety of mpox illness and care narratives from the stage of illness onset to nearly a year later.

### Narrating Acute Illness and Long-Term Effects

#### Acute Illness: From Mild to Severe

Participants described a range of experiences of mpox including minor symptoms and severe illness, and some participants reported hospitalisation (see Table 1 for a range of reported symptoms). The initial stages of illness were narrated in different ways: some participants described rapidly feeling unwell and symptoms such as fever and lesions, while others noticed initial symptoms that prompted them to attend a health service and then receive a diagnosis, with further symptoms developing later. Early illness was often described as one of profound sickness, although a few participants reported relatively minor symptoms. For example, Miller’s travel overseas to visit friends was cut short by infection, and he was subsequently hospitalised for 10 days due to severe symptoms. Miller remarked that “it was the worst pain I have ever experienced in my life” (initial interview). In contrast, while Matt recalled feeling initial distress due to diagnosis and isolation, he characterised his experience of illness as relatively benign: “I had it so mild, I feel so fortunate” (initial interview). Given the visual imagery of mpox circulating on social media, which often involved graphic pictures of people with many lesions, even participants who described feeling very unwell characterised themselves as ‘lucky’, as they had few lesions on visible parts of their body (particularly the face):

I was sort of expecting lesions to pop up everywhere. As it turned out, I only got the two lesions. So, I was one of the lucky ones. None on my face, none on any part of my body except the two that were next to my bum. (Daniel, initial interview)

**Table 1.** Participants’ Reported Acute Illness Symptoms and Long-Term Effects.

Participants	Acute illness symptoms	Long-term effects
Brady	Painless lesions (abdomen, anus), fatigue, fever	Nil reported
Calvin	Fever, rash, pain, painful lesions (torso), difficulty urinating and defecating, anorectal bleeding	Some remaining marks from lesions, but these cleared up over time
Daniel	Fever, headache, body aches, painful lesions (perianal), pain requiring hospitalisation	Anxiety and distress related to healthcare experience, receptive anal sex/bottoming disrupted for 6 months
David	Fever, painful lesions (face, wrist, legs, scrotum, anus), pain when defecating, anorectal discharge and bleeding, fatigue	Rectal bleeding lasted 4 months, surgery required for anal ulceration, no sex (5 months), some ongoing fatigue, abnormal heartbeat, some discolouration from scars
Johnny (one interview)	Painless lesions (pubic area and legs, elbow), night sweats	Nil reported
Leo (one interview)	Swollen groin, painless lesions (inner thigh)	Two sites where lesions have scarred (thigh), apprehensive about sex (feeling ‘dirty’)
Lucien	Painful lesions (face, arms, anus, ‘all over body’), secondary bacterial infection of lesions on the face, pain when defecating	Secondary bacterial infection took a month to heal. Scarring on the arms and face, one small scar on the face remains but others faded over time
Matt	Painless lesions (penis, leg, back), night sweats, fever, fatigue	Discolouration on scars but seemed to fade over time (not noticeable anymore)
Miller	Painful lesions (penis, anus, shoulders, palms of hands, soles of feet, arms, feet), sweating, fever, loss of appetite, difficulty urinating and defecating, inflamed foreskin, sepsis from infection, symptoms requiring hospitalisation	Scarring on penis and anus, ongoing proctitis (6 months), anus fistulated, multiple surgeries to correct gastrointestinal issues and remove anal skin tags. No sex life (10 months). Distress from interactions with healthcare
Paul	Painless lesions (in ‘first wave’: groin. In ‘second wave’: head, arms, and legs but not as severe as first)	Loss of interest in sex/erectile issues due to concerns about being contagious (3 months), distress from interactions with healthcare, some bouts of fatigue
Pete	Fever, painless lesions (penis), cough, pneumonia, body aches, fatigue, sweating, weight loss. Recommended hospitalisation but did not attend due to fears of stigma	Persisting cough (3 months), chronic fatigue (improving slightly over time), distress from interactions with healthcare
Ronald	Sinus congestion, body aches, headache, fatigue, painful lesions (anus, legs, back, finger), anorectal discharge and bleeding, loss of appetite, weight loss	Redness from lesions, eventually faded into slight discolouration. Some lumps and bumps around and inside anus
Tom	Itchy/painful anus, itchy leg, painful lesions (arms and legs)	Nil reported

There were common themes in participant accounts of acute illness: fears about lesions and scarring on visible parts of the body or on genitalia; relief at discovering that the disease was unlikely to be fatal; and a sense of frustration that clinical attention and health advice primarily focused on the evolution of lesions and not on other symptoms or on pain relief.

Many participants reported difficulties managing pain and other symptoms, including if they were unable to properly rest. For example, after being diagnosed with mpox, Calvin needed to keep working from home while managing illness, requiring him to switch between working and resting. His anal pain was particularly difficult:

It’s like constant thumping in my arse, random sudden onsets of searing intense pain where I’ve got to lie down and breathe. It’s 20 minutes at a time, and the pain never really goes away. But there were periods where it’s bearable and I could work. (Initial interview)

Calvin also found that anal pain made it difficult to urinate unless he had a hot shower, in which the heat appeared to relieve the pressure of his anal pain and allowed him to relax, involving multiple showers throughout the day. He also reported difficulties sleeping because of the pain, and he was prescribed sleeping medication, noting his doctor seemed reluctant to prescribe them.

In addition to being painful, the location of lesions – often on genitalia or around or inside the anus – was an additional source of distress for some participants. Lesions were usually near to sites of likely transmission [a pattern observed in Mitjà et al. (2023)], and participants reported feeling self-conscious about lesions and worried about the potential for stigma. Brady, who characterised his symptoms as mild and had a smaller number of lesions, explained:

It always felt a little bit salacious the way that people asked, “Oh, where are your blisters?” and I just didn’t really want to have that conversation. There’s nothing nice about having pus-filled scabby things around your butthole and I just didn’t want to have that conversation with anybody. […] There are some people who make being gay about having gay sex, like people look at [you] like you’re a sex act rather than who you have a relationship with. (Initial interview)

In addition to an invasion of privacy, Brady felt that voyeurism about symptoms reduced his personhood to his sexual practices, which might be treated with disgust or homophobia. The site of lesions (and possibility of scarring) ‘marked’ participants in a tangible way that could reveal their diagnosis to others, potentially opening them up to discrimination from others regarding their (assumed) sexual practices (Goffman, 1963/1990; Holt et al., 2010; Vytniorgu et al., 2022). Many participants were careful about who they disclosed their diagnosis to and only shared with close support networks. A few participants shared their story on social media aimed at other people in their community who may be at risk. These participants were motivated to destigmatise mpox and encourage their peers to take precautionary measures, such as seeking vaccination and temporarily reducing sexual practices to avoid mpox.

#### Long-Term Effects: “It’s Still Writing Its Story”

Participants were told by clinicians that scabbing of lesions and the revealing of healed skin beneath was regarded as the ‘end’ of the infectious period, usually taking several weeks. This occurred for almost all participants except for Paul, who experienced a second round of lesions, prolonging the acute illness and requiring further self-isolation totalling 1 month. While the disappearance of lesions marked an end to acute illness, many participants reported ongoing complications (see Table 1). While three participants reported a complete recovery with no symptoms after the acute illness period, others reported physical sequelae related to mpox (anorectal complications, scarring, and fatigue), along with ongoing effects on their mental health and sexual practices.

Miller experienced ongoing proctitis, anal ulcers, skin tags, and scarring from mpox which required multiple corrective surgeries. During his first interview, he was in between surgical procedures, and he described a persisting sense of living with mpox:

The pain and torture and the mindset has not changed. It’s something I’m still having to deal with. It’s not something I can put away and write a book about. It’s still writing its story. It’s been hard. (Initial interview)

At his follow-up interview, nearly 10 months after his initial infection, Miller reported that his anus was ‘normal’ again: “The last 10 days I’ve actually had no pain, no fear, and had control of my bowels. It’s been a very long road” (follow-up). During the preceding period, Miller regarded sex as ‘out of the question’. Daniel also reported a delay with sexual recovery, describing how it had taken 6 months before he was comfortable engaging in receptive anal sex to the same degree as prior to mpox.

People diagnosed with mpox in Australia were often informed that their semen could be infectious and to use condoms for oral and anal sex for up to 12 weeks (Communicable Diseases Network Australia, 2022). Some of the participants interpreted this as advice to pause sex completely, while some participants diagnosed overseas did not receive this advice. Paul reported a reduced interest in sex following the 12-week period. For example, he said, “I just started trying to be normal, but I was having erection issues. I had lost my sex drive. I had this overarching concern that I still had it in my system” (initial interview). Leo similarly reported an ongoing concern about infectivity and that he did not want partners to touch him.

The range of symptoms experienced in the acute stage were narrated by participants with confidence, because they appeared to match official accounts and reporting of cases. However, longer-term symptoms were described with a greater degree of uncertainty as participants were unsure whether their health problems were a result of mpox or if these symptoms were recognised in the medical literature as mpox sequelae. For example, David experienced fatigue and a slightly abnormal heartbeat for 5 months which he consulted a doctor about, revealing low iron levels. David explained, “I don’t know whether this is just something completely separate [to mpox]. I’ve not read anyone reporting anything like that” (initial interview). Pete reported an ongoing cough lasting 6 months and fatigue that persisted but improved over time. He was unsure if the fatigue was a result of the pneumonia he had experienced during mpox, as a result of the mpox virus itself, or a post-viral reaction: “No one has actually told me anything about what monkeypox does to you in the medium or long term” (follow-up). He described how fatigue had continued to affect him after acute mpox illness:

For about a month after I left isolation, it hits 3 pm and I’m in a ball, literally in a ball shaking, just extremely fatigued […] And the fevers and the body aches just still continuing, just going on and on and on. I call it my monkeypox wall. It eventually [shifted] from 3 pm to 4 pm, but now my wall is about 8pm and if I push through it, I feel quite better. (Initial interview)

At follow-up, Pete reported that his cough had ended, but fatigue was still present, if improved. As we address in a later theme, these varying long-term effects had impacts on some participants’ well-being and reflections on the acute illness event.

### Distressing Care (and Good Care as the Exception)

Amidst the vulnerable state of mpox illness, there was a common story shared by most participants. This involved negative experiences of care from health professionals and feeling judged about sexual behaviour within health services. As noted above, some participants characterised the pain relief they were provided for mpox as insufficient and some of the encounters they had with health professionals as causing distress. These participants felt that clinicians were not adequately addressing their concerns. When affirming experiences of care were described, these were almost always narrated in contrast to problematic encounters and as an exception rather than the norm.

#### Distressing Care

Beyond inadequate pain relief, some participants suggested clinicians (and health advice) had focused excessively on mpox lesions and the required isolation period rather than on symptoms that were causing more pain or concern to participants. For example, Pete, who was isolating at home in Australia and receiving phone calls from his sexual health doctor, explained:

Everything seems really standardised: you go into isolation, you wait for the lesion to dry up and leave and then you’re fine, but like what was happening to me, the last thing I was worried about was my lesion. (Initial interview)

Pete described his sexual health doctor asking him about the single mpox lesion which he had barely noticed, while Pete raised concerns about persistent coughing and coughing up blood:

I’m telling them “My cough is getting worse. My fever is getting worse.” And they were just like, “That’s just the virus progressing. You’ll be fine.” […] I was coughing up blood for about four days, and they’re like “Is it blood all day?” and I’m like, “No, mainly in the afternoons.” They’re like, “Okay. Is it speckled or is it pink?” I’m like, “It’s pink.” And they’re like, “Okay. You should be fine.” And I’m like, OK, this is normal then. (Initial interview)

After being told his symptoms were normal for several days, Pete was suddenly called by a hospital doctor and told an ambulance would be picking him up from home where he was isolating. Pete declined this option for several reasons: he felt his doctor had downplayed his symptoms and diminished his trust, he had not felt properly consulted about his care options, and he was not told how long he would be required to stay in hospital. He framed the hospital as a place that would not be as safe as his home and where he would be treated like an experiment:

“They’re like, ‘Oh you just have really interesting symptoms and so we really want to bring you in for a bunch of tests.’” His overall impression of the clinicians at this hospital was that “because I said ‘no’, they effectively just sort of washed their hands of me. They said, ‘Alright. Come in when you’re about to die’. They didn’t say that, they were just like ‘You’re being difficult’” (initial interview).

Pete clarified that he was willing to visit a health service as an outpatient (which he reported they would not permit) but did not feel comfortable being an inpatient with mpox.

Other participants reported problematic communications with health providers over the phone. Rocco (a close contact) reported feeling harassed by public health contact tracers, who repeatedly called him and implied that he and his housemate (who had been diagnosed with mpox) were in a sexual relationship. It was unclear to Rocco and his housemate why public health authorities had assumed they were in a relationship, which Rocco characterised as stereotyping about gay men. Despite not having spent time in the same room as his housemate since his housemate had returned from overseas, it took several days of arguing with public health staff over the phone to reclassify his risk level, as he was initially asked to isolate for several weeks:

It made me feel like I’m second class, like being gay is wrong again. […] It put me under a lot of stress because I was new in my role and I’m not out to my employers, I can’t say I’m going into isolation because why would you need to go into isolation for that long? They would figure, “Ah, it’s monkeypox. Well, you’re gay and you’re this and that.” I wasn’t ready for that. (Single interview)

Another participant, Tom, also experienced difficulties with contact tracing. Prior to his symptoms developing, he had been working in the office. After receiving his diagnosis, he was phoned by a contact tracer who requested a list of people who had been in the office to be notified anonymously as being close contacts. Tom explained what happened:

I probably went through every emotion possible for about 15 minutes before I then had the guts to call my boss and explain the situation. It was really tough to like basically declare it to HR and my boss and get this huge list together to match it up with all the phone numbers and then the [contact tracer] called back to say “he was very sorry that he had distressed me, but they decided not to pursue this” and I was extremely relieved, but I had been through a huge fear for my reputation. (Initial interview)

This miscommunication led Tom to disclosing his diagnosis to his employer, which fortunately resulted in support rather than discrimination, as Tom had feared. However, this was highly distressing on the same day as he had received his diagnosis.

The most distressed experiences of care occurred in hospitals. Daniel was initially diagnosed with mpox in an Australian hospital and, following discharge, found the pain relief he had been provided was insufficient. His symptoms worsened to the point that it felt like someone was ‘pouring acid inside’ his anus, requiring him to call an ambulance and return to hospital. Daniel reported that upon arrival to the hospital, he saw an emergency doctor who conducted an anorectal exam with inadequate consent:

He wanted to do a rectal exam on me and I was like, “I don’t want to have a rectal exam done. I’m in so much pain.” […] I was really reluctant because I really hadn’t consented to this whole thing, but he convinced me it was going to be fine. It was not fine. He didn’t wait the required amount of time with the local anaesthetic. He put it on and then 15 seconds later, he was like straight into me. I cried. My vision went to like tunnel vision. I was in so much pain. I was really unhappy. It seemed completely unnecessary. (Initial interview)

This event led to ongoing issues for Daniel. For example, he experienced “social anxiety, nightmares, flashbacks every time I’m near the hospital” (initial interview). Daniel’s mental health improved over time, after he spoke to a therapist. However, at follow-up, he reported a recent health emergency unrelated to mpox, in which he decided against calling an ambulance because he feared seeing the same doctor at the hospital.

Miller similarly engaged a psychologist to process his experiences with healthcare. In an overseas hospital, Miller reported highly stigmatising experiences of care:

They put me in a side room. They catheterised me with no pain relief, which was horrific. […] I got left in the side room for at least 6 hours, I was screaming in pain. I honestly would’ve rather died. It was almost like being an AIDS patient back in the ’80s. I felt so discriminated against. (Initial interview)

The comparison with AIDS here appeared to be a way to express the profound sense of injustice and neglect within a healthcare setting. In Miller’s case, this involved poor pain management, perceived judgement around his acquisition of mpox, and being isolated and neglected.

#### The Exception of Good Care

Miller stayed in hospital for 10 days and explained that he usually had to change his own sheets and that most staff would not remove rubbish from his room. However, there was one nurse who he trusted and who made his stay bearable:

She was the most gorgeous African lady who came in, held my hand, I cried with her, and I believe she said to me, “I’ve had family with HIV back in Africa and I understand,” and it’s only people with lived experience like that who I believe had a genuine understanding for how I felt and she was incredible, she really got me through. I was in hospital for 10 days, so she really got me through that. (Initial interview)

For Miller, this nurse’s actions and comparison of mpox with providing care for people living with HIV enabled him to feel safe with this one clinician. The narrative of ‘clinicians who made a difference’ amidst hostile, uncaring, or seemingly inept health systems appeared across multiple accounts. Having been diagnosed in Australia, Calvin characterised the doctor who diagnosed him as lacking in bedside manner but spoke about a public health nurse on the phone whose empathy made a huge difference shortly after receiving his diagnosis: “She just said ‘how are you?’ and I started to cry […] She said, ‘Look, this is not your behaviour. It’s a disease. It could be anyone’” (initial interview).

In telling his journey about navigating an overseas health system with his partner, Johnny remarked:

It wasn’t until we got to the infectious disease doctor who did a proper exam, and he honestly was the only one that acted like an actual doctor. He asked questions. He had gloves on, so he wasn’t afraid to touch me. The other ones seemed like they didn’t even want to get within breathing distance. (Initial interview)

For Johnny, ‘acting like a doctor’ involved appropriate examinations, proximity, and use of personal and protective equipment (PPE). Several participants reflected that clinicians appeared to be unprepared for working with a potentially contagious disease, which was surprising to them given the recency of COVID-19 and the normalisation of PPE. However, other participants reported that clinicians appeared to be comfortable and acknowledged the sense of alienation created by PPE: “Everybody was coming in there in PPE so I didn’t contaminate other spaces. So, I kind of knew instantly that they were taking it very seriously, but it was all very reassuring and done respectfully” (Tom, initial interview). These varied experiences suggest that subtle and simple aspects of communication shape how people interpret the quality of their care, and this is amplified in the context of a distressing illness (Gao et al., 2009; Waitzkin, 1984).

### Mpox as Socially Disruptive

In the above sections, we explored how mpox illness and care were narrated primarily as distressing experiences of poor health and poor care. Here, we turn to how mpox was narrated in its social effects, that is, how mpox reconfigured self, future, and participants’ normative values in relation to sex and prioritising time with friends and loved ones (Holt et al., 2010; Persson et al., 2019; Rosenfeld, 2006).

Participants grappled with the biographical meaning of an mpox diagnosis in different ways. Leo reflected that he previously did not regard sexually transmissible infections (STIs) as particularly serious or important but that mpox prompted him to reconsider his perspective to these infections and sex: “I’m just not as promiscuous as I was before. Like I’m very cautious now” (single interview). Other participants shared this sentiment: “It probably comes with age, but when something like [mpox] happens, it has an impact on your attitudes towards safer sex” (David, initial interview). A few participants evoked a much stronger moralistic framing. Tom observed:

Anything that we enjoy seems to have terrible consequences. Anything you enjoy is going to either kill you or make you sick. Everything in moderation, I guess. […] I’m not religious, but are these things sent to limit our behaviour and to scare us off, warn us, I don’t know. (Initial interview)

Here, mpox represented a threat of sexual excess, drawing on longstanding tropes that disease is a punishment for immorality (Persson et al., 2022; Sontag, 2002; Treichler, 1999). However, Tom’s conviction about this framing was simultaneously earnest and uncertain. Before the 2022 mpox outbreak, an easygoing and confident attitude towards sex had already been destabilised during COVID-19 lockdowns (Murphy et al., 2023). At follow-up, Tom described how his personal priorities around sex had shifted following the wake of these diseases:

I think between COVID and monkeypox combined, everybody has had an opportunity to rethink their priorities. I certainly will continue to be pretty sexually active, but I’m not going to drop everything for it like I probably did before. You know, dump friends on a night out because there’s a hook up. I’m not going to do that anymore. (Follow-up)

Other participants instead explicitly rejected any link between their mpox diagnosis and sexual behaviour and explained their diagnosis as a matter of bad luck, with mpox simply as a more unusual and serious STI.

In addition to challenging a sense of an easygoing sexual future, mpox also appeared to have troubled some participants’ self-confidence. Daniel described feeling disempowered:

It’s taken a long time for me to get really like happy and confident with sexual health. We’re brought up in this kind of world where we were told about HIV and how it kills you. And then overcoming a lot of that with PrEP and taking control of my own sexual health and feeling quite empowered, and then it feels like it kind of knocked me back a little bit. (Initial interview)

Here, the combined experience of ill health and care from mpox appeared to destabilise a sense of self-confidence that had been built up through growing up and dealing with the threat of HIV (Møller & Ledin, 2021). Lucien similarly observed that mpox revealed how positive experiences of healthcare for gay and bisexual men were contingent and precarious because they only exist in limited settings:

Overall the health system for queer people is quite amazing, you’ve got [local service], you’ve got PrEP. But as soon as you get into hospital with the main population, you’re not sure that people understand the queer situation. It sounds like we’re special, but we are special. And it worries me, because when you’re going through something painful and scary, you don’t want to have to educate people. (Follow-up)

In this account, Lucien’s trust in whether the health system might consistently and reliably support his community appeared to have been troubled.

While most participants dismissed the idea that mpox had changed them in a profound way, a few pointed to changes in their overall disposition towards life. Paul reported feeling isolated from other people, having reduced self-confidence, and that mpox had taken time away from his life, particularly as his two waves of lesions meant that he had to isolate for longer than others:

I feel like there’s been a portion of my life just cut away. It’s just this general feeling. I’m not a depressive natured person. I’d lost all this weight when I came back from overseas. Now it all has gone back on. […] I was stuck in my room for about 30 days. You know, it’s that actual drop from high to low and then trying to bring it back again. You try to engage with people you’ve been pushing away for a month. Like I’ve been pushing people away that I would normally catch up with and I’ve actually probably become more of a hermit than anything from it.

At follow-up, Paul reflected that the COVID-19 pandemic, mpox, and other life circumstances had combined to make him less enthusiastic about life.

A few other participants spoke about the quality of their relationships changing, demonstrating the ripple effects of illness in broader lifeworlds (Persson et al., 2019). Lincoln, a close contact, spoke about how it was challenging to go without physical touch from his partner for several weeks when they were sick and infectious with mpox: “I always knew that I enjoy touch and physical contact, but I didn’t realise how much not having it would have impacted me” (single interview). Pete’s ongoing fatigue also made it difficult for him to maintain a relationship with one of his partners:

It probably heavily contributed to my relationship issues. I couldn’t operate half the day, half the time. I was just so exhausted, and you can only expect people to have so much patience with you and hope that love sort of gets you through. But when it’s like, “no, I can’t sleep over, like I need to just actually lay here and not move and not be around people.” […] I sort of felt like she thought I was making up excuses when really, I was just struggling, and that took a really big toll. (Follow-up)

In contrast, for others the period of sickness and isolation was an opportunity to forge closer connections, as partners, friends, and family were there to provide support and comfort. For example, Lucien was staying with his sister overseas when he was diagnosed, requiring a longer stay due to the need to isolate. Her non-judgemental attitude during his illness had resulted in them having a stronger relationship which had been maintained since: “monkeypox helped in a sense that it kind of shoved us in this forced intimacy. It was the silver lining [to mpox]” (follow-up).

## Discussion

In this study, mpox was narrated as different kinds of disease: one involving both minor and severe illness, the effects of stigma, and an acute infection which sometimes had lasting consequences. These experiences were shaped by the conditions of ‘emergency’ and uncertainty that characterised the period of the 2022 outbreak. The unfamiliarity of mpox for health providers and community members, and the linkage of this outbreak with sexual transmission and gay and bisexual men, appeared to increase the difficulty of responding to and experiencing mpox.

As Rosenfeld (2006) has argued, both chronic and acute illnesses share the potential to disrupt self and social relationships. The concept of biographical disruption has most often focused on chronic illnesses (Bury, 1982; Wells et al., 2023), but as this study shows, the specific dynamics of acute illnesses warrant further exploration. Accounting for the variety of disruptive experiences engendered by a disease can offer valuable insight into the experience of illness and how institutions may recognise and respond to this disruptive potential (Bury, 2001; Hydén, 1997). Mpox is an interesting and unique case study of this given its rapid emergence as an outbreak in high-income countries in 2022 and the stigmatising media environment in which it appeared. Our findings illuminate both the extraordinary circumstances of the outbreak and its consequences for health and self, but also the ways in which illness can be disruptively mundane, manifesting in subtle and evolving changes to self and future.

While the symptoms of mpox were often a source of distress, the experiences in healthcare contexts appeared to compound the distress and long-term disruptive potential of mpox. The accounts in this study are reminiscent of the difficulties sexuality and gender diverse people experience accessing care from health services that lack competency with these populations (Apodaca et al., 2022; Newman et al., 2021; Smith, Davis et al., 2023). The disruption to sexual values and practices over time highlighted in this study were concerning, suggesting that mpox could have long-term impacts on sexual well-being that is not taken into consideration in short-term framings of outbreaks and acute infectious illness. Disruption to sexual well-being has long been a feature of diseases that are associated with sexual transmission, where ideas about blame, shame, and sexual morality come to the foreground of diagnosis (Cook, 2014; Holt et al., 2010; Persson et al., 2022). These disruptive qualities may require different forms and qualities of care.

Most participants reported perceived judgement and stigma regarding sexual behaviour from healthcare providers and a mismatch between patient needs and priorities (managing pain and discomfort) and clinical care (monitoring lesions and controlling transmission risk). Some participants drew on parallels with the response to HIV/AIDS in the 1980s, noting that care was inadequate or defensive and that patient-centred care was neglected. As Logie (2022) has argued, there are parallels between responses to HIV, COVID-19, and mpox in terms of stigma in the media and health settings. There were undoubtedly stressors on health services that may have contributed to poor care experiences (Garcia Iglesias et al., 2023; Hayes et al., 2024).

While public health responses are often focussed on containing, reducing, and eliminating onward transmissions, this study suggests that it is vital to also prioritise quality of patient care, especially during emergency contexts and where there is potential for compounding stigmatisation. The ‘positive’ instances of care narrated by participants involved staff explicitly challenging negative stereotypes and ideas about blame, acknowledging the distressing nature of the situation, and explaining why PPE was being used. In the context of a sexually transmitted disease, extra sensitivity is required, not only to challenge stigma but to also work in a way that is affirming and sensitive to the complex cultural and psychological elements entangled in sex and disease (Cook, 2014; Holt et al., 2010; Vytniorgu et al., 2022). Further, as Lancaster and Rhodes (2023) have argued, there is a need to attend to distal histories and long-term effects of outbreaks by taking a ‘slow’ view of outbreaks and ‘dis-ease’. For example, while most countries are now documenting much fewer new infections, the accounts in our study suggest that people who were diagnosed with mpox may continue to require support for scarring, rectal complications, and psychological distress related to experiences of care, even after the number of infections has reduced.

We note as a reflection that all qualitative interviewing is filtered through differing reconfigurations of memories (especially in longitudinal approaches like ours), the common cultural repertoires that are available to make sense of illness, and the co-construction of meaning within the interview encounter (Hydén, 1997; Mazanderani & Paparini, 2015). In our study, participants were making sense of their illness during periods of travel, within a noisy and stressful media environment, and many felt inadequately supported through their illness by health services. Indeed, the most unexpected part of this study was the striking similarities between participants’ accounts, in which they reported feeling neglected and vulnerable. Many participants also had limited opportunities to share their story with people in their lives (due to stigma) but wanted their experiences to count and improve experiences for others, motivating their participation in this research. The interviews were therefore often marked by a sense of urgency to share their story to an interviewer who was willing to listen. There were only a small number of people diagnosed with mpox in Australia, so our sample size reflects the outbreak and provides an important contribution as we were able to interview them 3 to 4 months after diagnosis. Moreover, we were able to do follow-up interviews with most participants to generate further insight into illness narratives and to document how mpox continued to have subtle effects in participants’ lives and sense of self. At the time of the follow-up interviews, many participants described feeling like they had ‘moved on’ from the distress of their illness event, and while they affirmed and reiterated the narratives from initial interviews, the mood of these interviews was vastly different, often characterised by less frustration. Participants may have found ways to reappraise and cope with the consequences of illness (Hagger & Orbell, 2022), but this shift also signals how meaning assigned to the illness event changes over time (Bury, 2001; Rosenfeld, 2006).

Given that our participants all experienced mpox during the height of the emergency outbreak in 2022, our study cannot illuminate how mpox may be experienced by people diagnosed or deemed a close contact at different times, or in different places, in which there may be reduced public attention (but also differing levels of preparedness or response). We also note that our sample were all white, cisgender men living in a high-income country. As Adetifa et al. (2023) note, mpox has impacted parts of Africa for at least five decades but has only received sustained global attention in the context of an outbreak impacting high-income countries. In the second half of 2023, most mpox cases have been notified in parts of the Western Pacific (World Health Organization, 2023a). Ethnographic and qualitative accounts of mpox in these different contexts are needed to inform health responses and global health inequalities.

## Conclusions

Attending to the experiences of acute illnesses offers insight into the disruptive qualities of disease for health and society. Mpox was a distressing illness experience, with some people in this study experiencing long-term consequences for health and well-being, along with subtle changes to self and future. Many healthcare encounters appeared to add to the distress of mpox, against the backdrop of a stigmatising media environment amidst the emergency outbreak. To our knowledge, this is one of the first in-depth qualitative explorations of mpox. These findings hold relevance for understanding illness narratives and disruption, and how care is prioritised in outbreak responses. Our study suggests that future public health responses to outbreaks need to better emphasise quality care, address both acute and potential long-term symptoms, and consider in more detail how stigma (re)produced in relation with sex and sexuality may shape experiences of illness. Healthcare services and systems would benefit from providing further training in cultural awareness and competence to provide appropriate and psychologically safe care for minority populations, including gay and bisexual men. Our findings also suggest that people previously diagnosed with mpox may continue to require other forms of care, including psychological care and support. This study is a timely reminder of our collective responsibilities to reject the idea that diseases are a moral failing and to take better care of those impacted by these stigmatising beliefs.

## Data Availability

Research data are not shared.
